# HEDGEHOG/GLI Modulates the PRR11-SKA2 Bidirectional Transcription Unit in Lung Squamous Cell Carcinomas

**DOI:** 10.3390/genes12010120

**Published:** 2021-01-19

**Authors:** Yiyun Sun, Dandan Xu, Chundong Zhang, Yitao Wang, Lian Zhang, Deqian Qiao, Youquan Bu, Ying Zhang

**Affiliations:** 1 Department of Biochemistry and Molecular Biology, Chongqing Medical University, Chongqing 400016, China; 2018110034@stu.cqmu.edu.cn (Y.S.); 2019110036@stu.cqmu.edu.cn (D.X.); zhangcd@cqmu.edu.cn (C.Z.); 190372@cqmu.edu.cn (Y.W.); 2018010003@stu.cqmu.edu.cn (L.Z.); 2020110030@stu.cqmu.edu.cn (D.Q.); buyqcn@cqmu.edu.cn (Y.B.); 2 Molecular Medicine and Cancer Research Center, Chongqing Medical University, Chongqing 400016, China

**Keywords:** hedgehog, GLI, PRR11, SKA2, lung squamous cell carcinoma

## Abstract

We previously demonstrated that proline-rich protein 11 (PRR11) and spindle and kinetochore associated 2 (SKA2) constituted a head-to-head gene pair driven by a prototypical bidirectional promoter. This gene pair synergistically promoted the development of non-small cell lung cancer. However, the signaling pathways leading to the ectopic expression of this gene pair remains obscure. In the present study, we first analyzed the lung squamous cell carcinoma (LSCC) relevant RNA sequencing data from The Cancer Genome Atlas (TCGA) database using the correlation analysis of gene expression and gene set enrichment analysis (GSEA), which revealed that the PRR11-SKA2 correlated gene list highly resembled the Hedgehog (Hh) pathway activation-related gene set. Subsequently, GLI1/2 inhibitor GANT-61 or GLI1/2-siRNA inhibited the Hh pathway of LSCC cells, concomitantly decreasing the expression levels of PRR11 and SKA2. Furthermore, the mRNA expression profile of LSCC cells treated with GANT-61 was detected using RNA sequencing, displaying 397 differentially expressed genes (203 upregulated genes and 194 downregulated genes). Out of them, one gene set, including BIRC5, NCAPG, CCNB2, and BUB1, was involved in cell division and interacted with both PRR11 and SKA2. These genes were verified as the downregulated genes via RT-PCR and their high expression significantly correlated with the shorter overall survival of LSCC patients. Taken together, our results indicate that GLI1/2 mediates the expression of the PRR11-SKA2-centric gene set that serves as an unfavorable prognostic indicator for LSCC patients, potentializing new combinatorial diagnostic and therapeutic strategies in LSCC.

## 1. Introduction

Lung cancer is one of the most common malignant tumors in the world. In China, lung cancer ranked first among malignancy for its incidence and mortality, and there has been a slight upward trend in its mortality rate over the past decade [1]. Approximately 85% of lung cancer cases are non-small cell lung cancer (NSCLC), with a predicted five-year survival rate of 15.9%. The two predominant NSCLC histological subtypes are lung adenocarcinoma (LADC, ~50%) and lung squamous cell carcinoma (LSCC, ~40%). LSCC is strongly associated with cigarette smoking, with characteristics of PIK3CA mutation, DDR2 mutation, FGFR1 amplification, and SOX2 amplification [2]. No LSCC-targeting therapeutic drugs are clinically available to date. Meanwhile, LADC-targeting therapies such as EGFR tyrosine kinase inhibitors and ALK inhibitors have minimal effect on treating LSCC [3]. Precision oncology aims to address inter-tumor heterogeneity through the armamentarium of drugs and the array of molecular targets [4]. However, more LSCC relevant genes need to be identified in order to decipher its mechanism and further improve their personalized diagnosis and therapy.

We previously identified proline-rich protein 11 (PRR11) as a novel NSCLC-related gene that is implicated in the cell cycle, cytoskeleton remodeling, and metastasis [5,6,7]. Moreover, we demonstrated that PRR11, along with its upstream adjacent gene (spindle and kinetochore associated 2 (SKA2)), constitutes a classic head-to-head gene pair driven by a prototypical bidirectional promoter containing CCAAAT boxes. SKA2 is also a putative tumor-related gene whose protein product is involved in chromosome segregation and cell cycle regulation. The PRR11-SKA2 bidirectional transcription unit is the target of the transcription factors P53 and NF-Y, cooperatively accelerating NSCLC development. Furthermore, NSCLC patients with high expression of both PRR11 and SKA2 show poorer prognosis compared to all other patient groups [8,9]. Hence, we hypothesize that the PRR11-SKA2 gene pair rather than a single gene would potentially become an effective target in the diagnosis and treatment of NSCLC. Nevertheless, the signaling pathways responsible for the ectopic expression of this gene pair and thus malignant phenotypes are unknown, and need further exploration.

Hedgehog (Hh) signaling is a conserved pathway that directs embryonic development via the temporal and spatial regulation of cellular proliferation and differentiation [10]. In the canonical Hh pathway, upon binding of a Hh ligand (SHH, IHH, and DHH), the transmembrane receptor PATCHED (PTCH) relieves its inhibition on the G protein-coupled receptor SMOOTHENED (SMO), which in turn activates the downstream GLI transcription factors (GLI1, GLI2, and GLI3) to modulate the transcription of target genes. Both GLI1 and GLI2 mainly act as transcriptional activators, and several components of the Hh pathway, such as PTCH1, GLI1, and GLI2, are also GLI transcriptional targets. Apart from being stimulated by the canonical Hh-PTCH-SMO route, GLI1 and GLI2 are activated through non-canonical mechanisms, which are SMO-independent but determined by other pathways, including PI3K-AKT, RAS-MEK, and TGF-β, etc. [11]. The Hh pathway mediates the interplay between mesenchymal and epithelial cells, which is vital for the branching morphogenesis of embryonic lungs [12]. In general, the Hh pathway becomes inactivated after lung maturation. However, there is a growing body of literature pointing to aberrant activation of Hh pathway in lung cancer, especially LSCC. Shi’s lab conducted an integrative analysis of three LSCC microarray datasets and found that LSCC exhibited Hh signaling activation [13]. A multitude of clinical data also attests to the dysregulation of the Hh pathway in LSCC [14,15,16,17,18]. It is worth noting that the reactivation of the Hh pathway in LSCC is probably ascribed to smoking, which is an important risk factor for LSCC. Basbaum’s lab demonstrated that cigarette-smoke induced the Hh pathway in human primary or immortalized bronchial epithelial cells and thus elicited tumorigenesis in these cells [19]. Several lines of evidence suggest that the Hh pathway plays an important role in NSCLC tumorigenesis via regulating stemness features [20,21,22,23,24], drug resistance [25,26,27,28], epithelial-mesenchymal transition, invasion, migration [16,29,30,31,32,33,34], and cellular proliferation [17,31,32,33,35,36,37,38,39]. Taking a close relationship between the Hh pathway and LSCC into consideration, we considered whether Hh signaling served as an upstream pathway to modulate the expression of the PRR11-SKA2 gene pair. In this study, we present the first evidence that the non-canonical Hh pathway could promote the expression of the PRR11-SKA2-centric gene set (including PRR11, SKA2, BIRC5, NCAPG, CCNB2, and BUB1), potentializing new combinatorial diagnostic and therapeutic strategies in LSCC.

## 2. Materials and Methods

### 2.1. Gene Set Enrichment Analysis (GSEA)

The RNA sequencing data of LSCC, including 500 tumor tissues and 49 normal lung tissues were downloaded from The Cancer Genome Atlas (TCGA) database [40]. The Pearson correlation test was adopted for the correlation analysis of gene expression between PRR11 or SKA2 and other genes [41]. GSEA (http://software.broadinstitute.org/gsea/downloads.jsp) was applied to the PRR11-SKA2 correlated gene list to explore other similar gene sets [42]. In detail, the ‘GSEA Preranked’ module and the ‘C6.all.v6.1.symbols.gmt [oncogenic signatures]’ geneset database were chosen. Further, ‘1000 gene of permutations’ and ‘no collapse to gene symbols’ were set. Nominal *p*-value < 0.05, FDR < 0.25, and gene set size >100 were defined as the cut-off criteria.

### 2.2. Cell Culture and Treatment

The human lung squamous cell carcinoma cell lines H226, H1270, and SK-MES-1 were obtained from the Cell Bank of Type Culture Collection of the Chinese Academy of Sciences (Shanghai, China). The human lung squamous cell carcinoma cell line H520 was purchased from Kunming Cell Bank of Type Culture Collection (Kunming, China). Cells were maintained in RPMI 1640 medium (H520, H226, and H2170) or high glucose-DMEM (SK-MES-1) supplemented with 10% fetal bovine serum (Invitrogen, CA, USA), penicillin (100 IU/mL), and streptomycin (100 μg/mL). All cells were cultured in a humidified incubator at 37 °C with 5% CO_2_.

For drug treatment studies, GANT-61 (Sigma-Aldrich, St. Louis, MO, USA), GDC-0449 (Selleckchem, Houston, TX, USA), and SAG (Santa Cruz Biotechnology, Dallas, TX, USA) were prepared in DMSO. Cells were treated with either DMSO control or drugs at designated concentrations for 24 h or 48 h. These cells were then collected and subjected to analysis.

### 2.3. siRNA Transfection

GLI1-targeting and GLI2-targeting siRNAs were chemically synthesized via Shanghai GenePharma (Shanghai, China), which were previously described [43,44]. The sense sequences of these siRNAs included GCCACCAAGCUAACCUCAUGUTT (GLI1-siRNA), CCUUCAAGGCGCAGUACAUTT (GLI2-siRNA), and UUCUCCGAACGUGUCACGUTT (NC-siRNA). The transient transfection of the indicated cells with siRNAs was conducted using Lipofectamine^TM^ RNAiMAX reagent (Invitrogen, CA, USA) according to the manufacturer’s instructions. These cells were then collected and subjected to analysis 72 h post-transfection.

### 2.4. Cell Proliferation Assay

Cells were seeded in 96-well plates at a density of 1000 cells per well and incubated with the complete medium. After adherent growth, cells were changed into the medium with either DMSO or the designated concentrations of GANT-61 and treated for 6 days. Cell proliferation was measured by Cell Counting Kit-8 (Biomaker, Austin, TX, USA), according to the manufacturer’s instructions. The viable cells at each time point were presented as the corresponding absorbance at 450 nm. Three independent experiments were performed in triplicates.

### 2.5. Colony Formation Assay

Cells were seeded in 6-well plates at a density of 1000 cells per well and incubated with a complete medium. After adherent growth, cells were changed into the medium with either DMSO control or the designated concentrations of GANT-61 and treated for around 14 days. Then cells were sequentially washed twice with PBS, fixed with 4% paraformaldehyde for 15 min, stained with 0.2% crystal violet solution for 30 min, and washed three times with PBS before images were obtained. Every experiment was performed in triplicates. Three independent experiments were performed in triplicates.

### 2.6. Quantitative RT-PCR

Total RNA was isolated from cells using the TRIzol^TM^ Reagent (Invitrogen, Carlsbad, CA, USA). The cDNA was synthesized from 500 ng of total RNA using the PrimeScript1^st^ Strand cDNA Synthesis Kit (Takara, Tokyo, Japan) following the manufacturer’s instructions. Quantitative real-time PCR was performed through the SYBR PremixEx Taq^TM^ (Takara, Tokyo, Japan). The relative expression levels of the target genes were calculated with the 2^−△△Ct^ method. The sequences of the primers used are provided in Table S1.

### 2.7. Western Blot

The Western blot procedure has been previously described in detail [7]. The primary antibodies used were mouse monoclonal anti-PRR11 (1:1000) (TA800449, Origene, MD, USA), rabbit polyclonal anti-SKA2 (1:500) (PA5-20818, Invitrogen, Waltham, CA, USA), rabbit polyclonal anti-GLI1 (1:1000) (2534, Cell Signaling Technology, Danvers, MA, USA), rabbit polyclonal anti-GLI2 (1:400) (ab26056, Abcam, Cambridge, MA, USA), rabbit polyclonal anti-NF-Y (1:1000) (sc-13045X, Santa Cruz Biotechnology, Dallas, TX, USA), and rabbit polyclonal anti-GAPDH (1:5000) (AB-P-R001, Goodhere Biotechnology, Hangzhou, China).

### 2.8. RNA Sequencing

H226 cells were treated with either 30 μM GANT-61 (Sigma-Aldrich, St. Louis, MO, USA) or DMSO control for 48 h, then the total RNA was extracted from cells using TRIzol^TM^ Reagent (Invitrogen, Carlsbad, CA, USA). After quantitative analysis and quality inspection, the RNA samples were sent to Novogene (Beijing, China) for RNA sequencing. Briefly, NEBNext^®^ Ultra^TM^ RNA Library Prep Kit for Illumina^®^ (New England Biolabs, Ipswich, MA, USA) was used to construct sequencing libraries. Each sample was subjected to DNA sequencing with the Illumina NovaSeq6000 Sequencing System. The raw data was then preprocessed and normalized. The RNA sequencing dataset was deposited in the Gene Expression Omnibus (GSE161226).

### 2.9. Identification and Bioinformatic Analysis of Differentially Expressed Genes

To identify the differentially expressed genes in the GANT-61 group compared to the DMSO control group, |Log2(Fold change)| > 1 and padj < 0.05 were set as the cut-off criteria. Functional enrichment in gene ontology (GO) terms was performed using the clusterProfiler R package [41], and the protein-protein interaction network was analyzed using the String database for Homo sapiens [45].

### 2.10. Statistical Analysis

All statistical analyses were carried out using the SPSS software, version 17.0 (SPSS Inc., Chicago, IL, USA). Comparisons between two groups were performed via the independent Student’s *t*-test, and correlations between gene expression levels were evaluated using the Pearson correlation test. All comparisons were two-tailed, and *p* values of <0.05 were taken to be statistically significant. The LSCC-related gene expression dataset (GSE4573) was downloaded from the Gene Expression Omnibus (GEO) database and further used for the Kaplan–Meier survival analysis [46]. The receiver operating characteristic curve analysis was conducted to identify a rational cut-off point. In brief, patients were divided into a good prognostic group and poor prognostic group according to the five-year overall survival period, then the cut-off point that reached optimal sensitivity and specificity was selected. Subsequently, the high-expression group and the low-expression group were classified based on the cut-off point and then analyzed by log-rank test.

## 3. Results

### 3.1. The Expression of the PRR11-SKA2 Gene Pair Significantly Correlates with the Hh Pathway in LSCC

**Figure 1:**
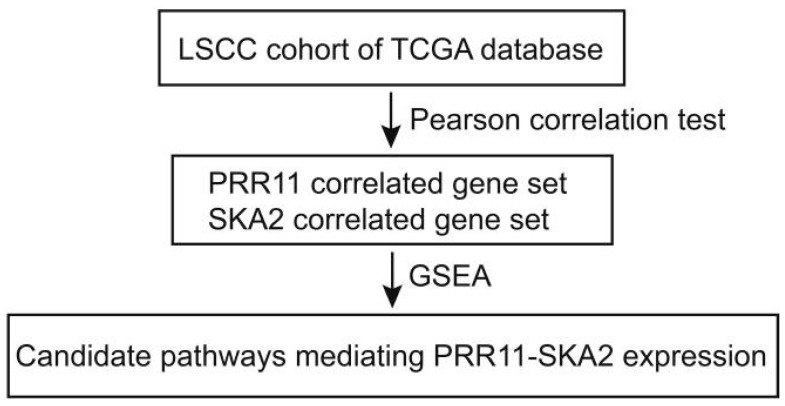
Analysis pipeline of the PRR11-SKA2 correlated gene set in the LSCC cohort of TCGA. LSCC, lung squamous cell carcinoma; TCGA, The Cancer Genome Atlas; PRR11, proline-rich protein 11; SKA2, spindle, and kinetochore associated 2.

**Figure 2:**
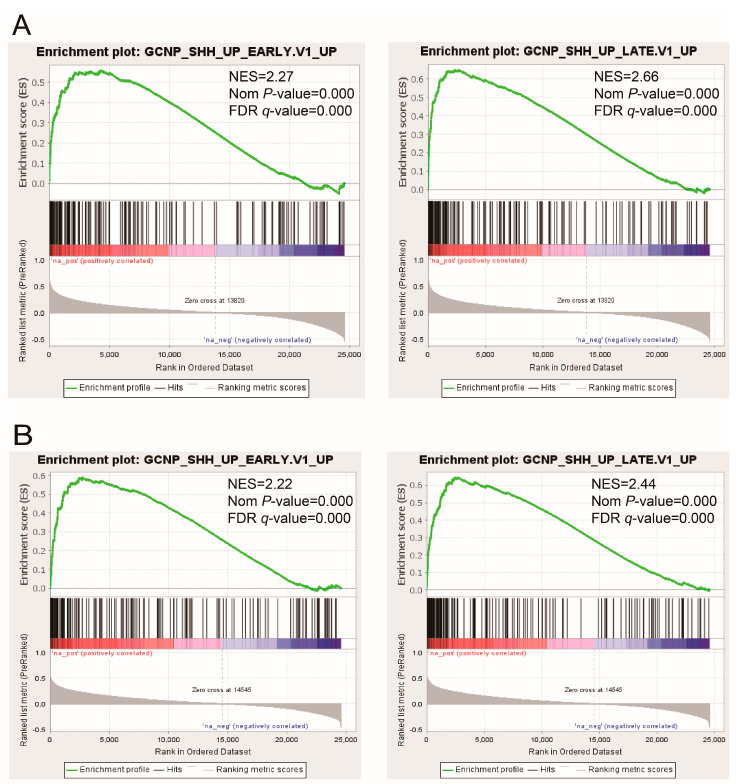
GSEA of the PRR11-SKA2 correlated gene set in the LSCC cohort of TCGA. ‘GCNP_SHH_UP_EARLY.V1_UP’ and ‘GCNP_SHH_UP_LATE.V1_UP’ gene sets were enriched in both the PRR11 positively correlated gene list (**A**) and the SKA2 positively correlated gene list (**B**). The normalized enrichment score (NES), the nominal *p*-value, and the false discovery rate (FDR) were calculated for each gene set. Nominal *p*-value < 0.05 and FDR *q*-value < 0.05 were taken to be statistically significant.

**Figure 3:**
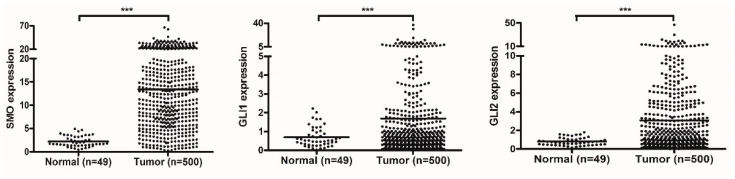
The Hh pathway components are overexpressed in the LSCC cohort of TCGA. Scatter plots of gene expression levels of the Hh pathway components (SMO, GLI1, and GLI2) in normal tissues and tumor tissues are displayed from left to right. Mean values are represented by horizontal lines (two-sided independent Student’s *t*-test, *** *p* < 0.001).

The LSCC RNA sequencing data were downloaded from the TCGA database and then employed in the correlation analysis of gene expression, further creating two gene lists highly correlated with PRR11 expression and SKA2 expression, respectively (Table S2). To provide further insight into oncogenic signaling pathways mediating the expression of the PRR11-SKA2 gene pair, GSEA was conducted to explore the two aforementioned gene lists based on ‘C6: oncogenic signatures gene sets’ (Figure 1 and Table S3). As a result, two gene sets were remarkably enriched in both the PRR11 positively correlated gene list and the SKA2 positively correlated gene list, namely ‘GCNP_SHH_UP_EARLY.V1_UP’ and ‘GCNP_SHH_UP_LATE.V1_UP’ (Figure 2). It implied that the PRR11-SKA2 correlated gene list extremely resembled the Hh pathway activation-related gene set. Moreover, based on the LSCC RNA sequencing data from the TCGA database, the expression levels of Hh pathway-related positive components (including SMO, GLI1, and GLI2) in LSCC tumor tissues were significantly higher than those in normal lung tissues (Figure 3), reflecting the LSCC characteristic of Hh pathway activation. As indicated from these results, the high expression of the PRR11-SKA2 gene pair in LSCC was probably attributed to an activated Hh pathway.

### 3.2. GLI1/2 Modulates the Expression of PRR11 and SKA2 in LSCC Cells

**Figure 4:**
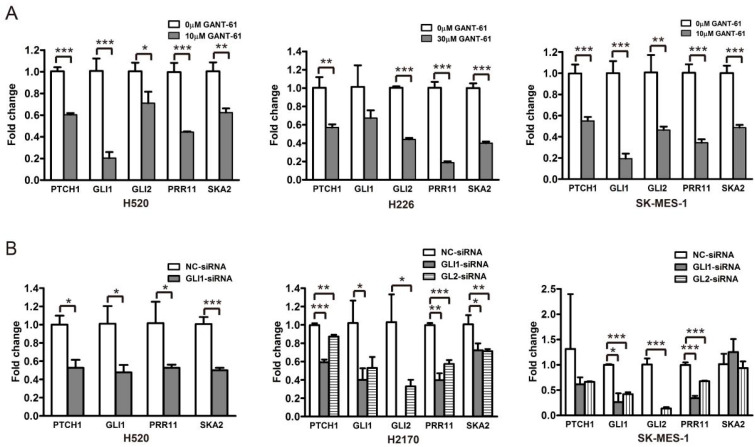
GLI1/2 modulates the expression of PRR11 and SKA2 in LSCC cell lines. (**A**) The expression of the Hh pathway components (PTCH1, GLI1, and GLI2), PRR11 and SKA2 in LSCC cells (H520, H226, and SK-MES-1) treated with GANT-61 was measured using quantitative RT-PCR. (**B**) The expression of the Hh pathway components (PTCH1, GLI1, and GLI2), PRR11 and SKA2 in LSCC cells (H520, H2170, and SK-MES-1) transfected with the GLI1-siRNA or GLI2-siRNA was measured using quantitative RT-PCR. GAPDH served as the loading control. Three independent experiments were performed in triplicates and data are shown as the mean ± SD (two-sided independent Student’s *t*-test, * *p* < 0.05, ** *p* < 0.01, and *** *p* < 0.001). Note that the data for GLI2-siRNA for H520 cells is not included due to the weak knockdown efficiency of GLI2-siRNA in H520 cells.

**Figure 5:**
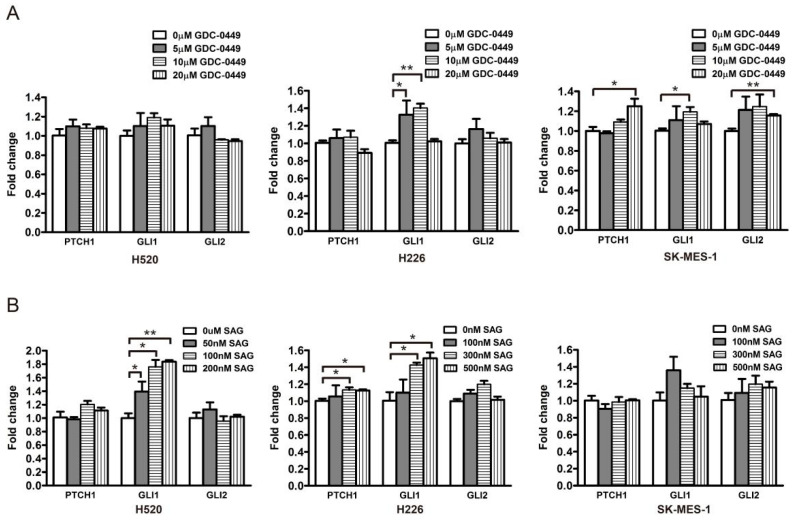
SMO does not modulate the expression of GLI target genes in LSCC cells. GDC-0449 is an SMO antagonist and SAG is an SMO agonist. (**A**) The expression of the Hh pathway components (PTCH1, GLI1, and GLI2) in LSCC cells (H520, H226, and SK-MES-1) treated with GDC-0449 was measured by quantitative RT-PCR. (**B**) The expression of the Hh pathway components (PTCH1, GLI1, and GLI2) in LSCC cells (H520, H226, and SK-MES-1) treated with SAG was measured using quantitative RT-PCR. GAPDH served as the loading control. Data are shown as the mean ± SD (two-sided independent Student’s *t*-test, * *p* < 0.05 and ** *p* < 0.01).

To corroborate the relationship between the Hh pathway and its potential downstream effectors, i.e., PRR11 and SKA2, three LSCC cell lines (H520, H226, and SK-MES-1) were treated with a GLI1/2 inhibitor, GANT-61, and a SMO inhibitor, GDC-0449, respectively, and then assayed for morphologic and molecular alterations. Compared with the control, GANT-61 significantly reduced the expression levels of Hh pathway downstream targets (PTCH1, GLI1, and GLI2), confirming the inhibition of Hh signaling (Figure 4A and Figure S1). Moreover, GANT-61 treatment remarkably decreased the expression levels of PRR11 and SKA2 (Figure 4A and Figure S1), corresponding to the strong suppression of cell proliferation and colony formation (Figure S2). In addition, siRNAs targeting GLI1 or GLI2, separately denoted as GLI1-siRNA and GLI2-siRNA, were utilized to suppress the Hh pathway in three LSCC cell lines (H520, H2170, and SK-MES-1). Consistently, the knockdown of GLI1 or GLI2 also declined the mRNA levels of PRR11 and SKA2 (Figure 4B). It is noteworthy that neither the SMO antagonist GDC-0449 nor the SMO agonist SAG could effectively change the mRNA levels of the Hh pathway downstream targets (Figure 5), implying a minimal role of SMO in mediating the Hh pathway in LSCC. In agreement with this idea, GDC-0449 had minor effects on the survival of LSCC cell lines relative to GANT-61 (Figure S3). Taken together, these findings suggest the expression of the PRR11-SKA2 gene pair is regulated via non-canonical Hh signaling, which is dependent on GLI1/2 rather than SMO.

### 3.3. GLI1/2 Mediates the Expression of Other Genes Correlated with the PRR11-SKA2 Gene Pair in LSCC Cells

**Figure 6:**
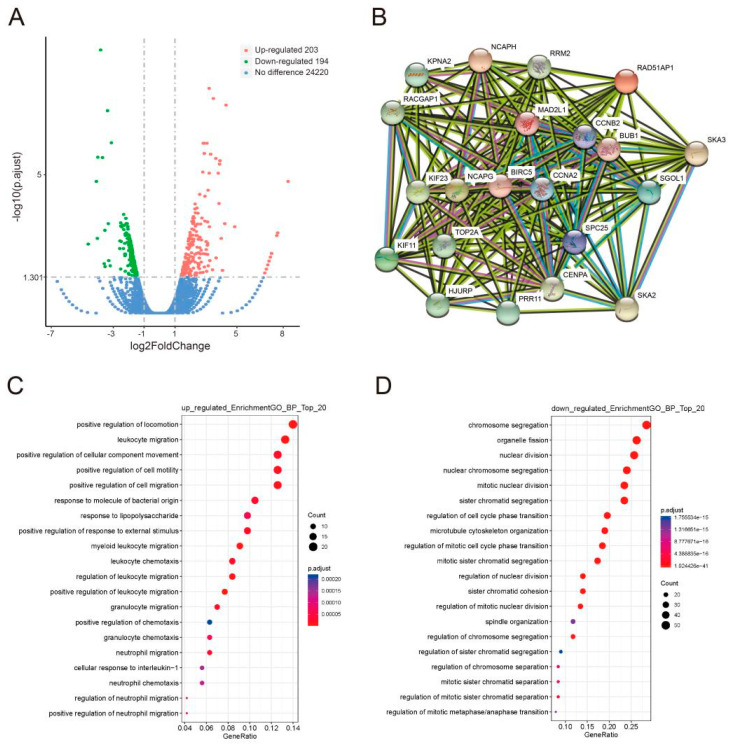
Identification and functional categories of differentially expressed genes associated with GLI1/2 inhibition. (**A**) Volcano plot of the gene expression profile. The red dots and the green dots represent upregulated genes and downregulated genes, respectively. (**B**) The protein–protein interaction network of 19 downregulated genes relating to the PRR11-SKA2 gene pair was built via the String database. (**C**,**D**) GO enrichment analysis of differentially expressed genes. The top 20 biological processes enriched in the upregulated genes (**C**) and the downregulated genes (**D**) are shown on the *y*-axis.

To unravel those genes whose expression levels are correlated to the PRR11-SKA2 gene pair, as well as directed by the Hh pathway, LSCC cells were treated with GANT-61 or DMSO, and then the corresponding mRNA expression profiles were detected via RNA sequencing (Table S4). A total of 397 differentially expressed genes were identified in the GANT-61 group relative to the control group, including 203 upregulated and 194 downregulated genes (Figure 6A). Furthermore, GO enrichment analysis revealed that the prevalent biological process categories among the upregulated genes were movement relevant GO terms, such as ‘leukocyte migration’, ‘neutrophil migration’, ‘leukocyte chemotaxis’, and ‘neutrophil chemotaxis’ (Figure 6C). In contrast, the downregulated genes were mainly classified in cell division relevant GO terms, such as ‘chromosome segregation’, ‘nuclear division’, ‘regulation of cell cycle phase transition’, and ‘spindle organization’ (Figure 6D).

**Figure 7:**
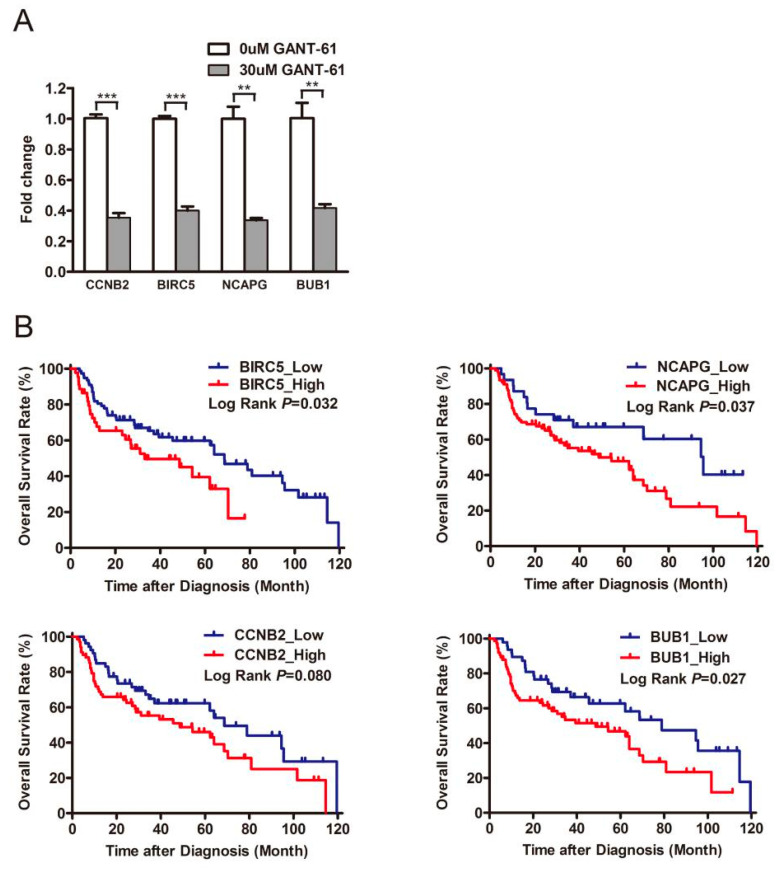
Validation and Kaplan–Meier survival analysis of differentially expressed genes. (**A**) The expression of BIRC5, NCAPG, CCNB2, and BUB1 in H226 cells treated with GANT-61 was measured using quantitative RT-PCR, with GAPDH as the loading control. Data are shown as the mean ± SD (two-sided independent Student’s *t*-test, ** *p* < 0.01 and *** *p* < 0.001). (**B**) Survival analysis of four crucial genes (BIRC5, NCAPG, CCNB2, and BUB1) in LSCC. Kaplan–Meier plots of overall survival of LSCC patients are based on the GSE4573 dataset. The survival curves are compared using log-rank test. *p*-values are two-sided and *p* < 0.05 is considered statistically significant.

As previously described, the gene set correlated with the PRR11-SKA2 gene pair was created based on the LSCC RNA sequencing data from the TCGA database (|correlation coefficient| > 0.5). This gene set included 19 genes overlapping with the aforementioned 194 downregulated genes (Table S5). Subsequently, the protein–protein interaction network of these 19 genes and the PRR11-SKA2 gene pair was built by String database, clustering on a “cell division” subnetwork (Figure 6B). Out of this subnetwork, BIRC5, NCAPG, CCNB2, and BUB1 interacted with both PRR11 and SKA2. The following validation of these four genes, via quantitative RT-PCR, showed that they were significantly downregulated in LSCC cells treated with GANT-61 (Figure 7A), coinciding well with the previous RNA sequencing results. As noted, PRR11 and SKA2 are candidate prognostic indicators for NSCLC whose expression levels are negatively correlated with overall survival [7,9]. Therefore, one LSCC relevant GEO dataset was applied to further explore the relationship between these four crucial genes and the survival of LSCC patients. Analogously, high expression of BIRC5 (*p* = 0.032), NCAPG (*p* = 0.037), CCNB2 (*p* = 0.080) or BUB1 (*p* = 0.027) remarkably correlated with shorter overall survival for LSCC patients (Figure 7B). These findings reflect that GLI1/2 directs the expression of the PRR11-SKA2-centric gene set (including PRR11, SKA2, BIRC5, NCAPG, CCNB2, and BUB1), which potentially acts as a combinatorial prognostic indicator for the overall survival of LSCC patients.

## 4. Discussion

In our previous work, the PRR11-SKA2 bidirectional transcription unit was identified as an NSCLC-associated “head-to-head” gene pair regulated by the transcription factors P53 and NF-Y through binding to the bidirectional promoter of this gene pair. Furthermore, this gene pair synergistically promoted the development of NSCLC [8,9]. In order to unravel the signaling pathways accounting for the ectopic expression of the PRR11-SKA2 gene pair in LSCC, the LSCC RNA sequencing data from the TCGA database was consecutively dissected by the correlation analysis of gene expression and GSEA, revealing the high similarity between the PRR11-SKA2 correlated gene list and the Hh pathway activation-related gene set (Figure 2). This suggests that the Hh pathway presumably drives the high expression of the PRR11-SKA2 gene pair. In the following experiments, GLI1/2 inhibitor GANT-61 or GLI1/2-siRNA inhibited the Hh pathway and concomitantly decreased the expression levels of PRR11 and SKA2 in LSCC cell lines (Figure 4), clarifying that the Hh pathway serves as the upstream signaling pathway to modulate the PRR11-SKA2 gene pair. As the main mediators of the Hh pathway, the transcription factors GLI1 and GLI2 directly control the transcription of target genes via binding to the consensus sequence (GACCACCCA) in their promoters [47]. Bioinformatic analysis does not identify these canonical GLI-binding sites in the PRR11-SKA2 bidirectional promoter region, despite this region containing other potential transcription factor binding sites, such as NF-Y, P53, NF-κB, MYB, E2F1, and SP1 [9]. Hence, it lacks direct evidence with respect to the issue of how GLI1/2 affects the expression of this gene pair. As described above, we reported that NF-Y increased the expression of the PRR11-SKA2 gene pair through directly binding to their bidirectional promoter region. We thus put forward one possible mechanism that NF-Y is one transcriptional target of GLI1/2 and GLI1/2 prompt the gene pair expression through NF-Y. In order to corroborate this, the expression level of NF-Y in LSCC cells (H520 and H226) treated with GANT-61 was detected by WB, and the result showed that GANT-61 remarkably reduced the expression level of NF-Y in LSCC cells (Figure S4). Given there are no canonical GLI-binding sites in the NF-Y promoter region, we speculate that the NF-Y promoter region harbors variant GLI-binding sites, which needs to be verified in future work.

The canonical Hh pathway unambiguously displays the Hh-PTCH-SMO-GLI route, and its aberrant activation has been shown in a variety of cancers, including basal cell carcinoma, gliomas, medulloblastoma, leukemias, breast cancer, lung cancer, and pancreatic cancer, etc. To date, two SMO inhibitors (LDE-225/Sonidegib and GDC-0449/Vismodegib) and one GLI2 inhibitor (arsenic trioxide, ATO) have received the US Food and Drug Administration (FDA) approval for treating basal cell carcinoma and acute promyelocytic leukemia, respectively [48]. Distinct from the canonical Hh pathway, the non-canonical Hh pathway exerts signaling in three ways. First, the binding of Hh and PTCH does not affect SMO, but disrupts the interaction of PTCH with cyclin B1 and the proapoptotic complex, thereby leading to increased cellular proliferation and survival. Second, the SMO signaling does not act on GLI. Instead, SMO regulates the actin cytoskeleton through RhoA and Rac1, or stimulates calcium release from the endoplasmic reticulum via the PLC-IP3 pathway [49]. Third, GLI is activated by other signaling pathways rather than the Hh ligand or PTCH1/SMO. The involved pathways are mostly oncogenic, such as PI3K-AKT, RAS-MEK, and TGF-β [11]. It was initially believed that NSCLC substantially exhibited the activation of the canonical Hh pathway [13,14,16,19,26,27,29,32,34,35,39,50]. Nevertheless, emerging evidence also indicates that the non-canonical Hh pathway outperforms the canonical one in NSCLC [17,22,23,36,38,51]. Onaitis’s lab found that the SMO-shRNA and the SMO inhibitor GDC-0449 had minor effects on the expression levels of GLI transcriptional targets, cellular proliferation, and survival, whereas the GLI2-shRNA and the GLI2 inhibitor GANT-61 were very effective in LSCC [17]. Consistent with their findings, we observed that neither the SMO inhibitor GDC-0449 nor the SMO agonist SAG altered the expression levels of GLI transcriptional targets in LSCC cells (Figure 5). In contrast to GANT-61, GDC-0449 hardly impacted cell survival (Figure S3). The inefficiency of SMO-targeting regimens is ascribed to the complexity of the Hh pathway. For instance, LSCC cells carry SMO mutations, which mitigate the efficiency of SMO agonists and antagonists. Several SMO mutations, such as G497W and D437H, have been uncovered as actors in GDC-0449 resistance [11]. Additionally, as noted, there is the SMO-independent non-canonical Hh pathway plays a role, as well. PI3K-AKT and MAPK-ERK pathways could directly activate GLI1 in LSCC [38]. Given that the Hh pathway and other oncogenic pathways converge to activate GLI, a combinatorial therapeutic strategy that targets GLI and other pathways involved in GLI activation are rational to address the resistance to SMO inhibitors.

In fact, we found that GLI1/2 regulated the expression of the PRR11-SKA2-centric gene set in LSCC cells, including PRR11, SKA2, BIRC5, NCAPG, CCNB2, and BUB1, which determines cell division as well as the prognosis of LSCC patients (Figure 7). BIRC5 (also known as Survivin) is one component of the chromosome passenger complex (CPC), directing CPC movement to different locations during cell division. In general, BIRC5 favors cellular proliferation and represses cell apoptosis. BIRC5 has been demonstrated as the target gene of GLI2 [52]. BIRC5 vaccines for cancer immunotherapy have successfully passed proof-of-concept and have already been applied in clinical trials to treat malignant glioblastoma, ovarian cancer, breast cancer, colon cancer, lung cancer, and the like [53,54,55,56]. NCAPG is the regulatory subunit of the condensin complex, which is required for the conversion of interphase chromatin into a condensed chromosome. NCAPG is highly expressed in a variety of tumors, serving as a promising therapeutic target [57]. CCNB2 is essential for the control of the cell cycle at the G2-M transition. Other studies have identified CCNB2 as a hub protein in the NSCLC protein-protein interaction network, and its overexpression is an unfavorable prognostic factor for NSCLC patients [58,59,60]. BUB1 is a central component of the mitotic checkpoint for spindle assembly, mediating cell death in response to chromosome missegregation and thus suppressing spontaneous tumorigenesis [61]. Although BUB1 seems to be a tumor suppressor, enhanced BUB1 expression causes chromosome instability and further induced oncogenesis [62]. Similar to CCNB2, BUB1 is also a hub protein in the NSCLC protein-protein interaction network and its overexpression is a poor prognostic indicator for NSCLC patients [59,63]. BUB1 inhibitors, in combination with taxanes or PARP inhibitors, could improve treatment efficacy and potentially overcome drug resistance [64]. Together, GLI1/2 and this PRR11-SKA2-centric gene set might be considered when designing combinatorial therapeutic regimens for LSCC.

## 5. Conclusions

In summary, we demonstrated that Hh-GLI signaling regulates the expression of PRR11-SKA2-centric gene set in sLSCC. Our findings not only shed light on the molecular mechanism that gives rise to the overexpression of the PRR11-SKA2 gene pair in LSCC but also support the basis of the new idea clustering on the PRR11-SKA2 gene pair in LSCC personalized diagnosis and therapy.

## Supplementary Materials

The following are available online at https://www.mdpi.com/2073-4425/12/1/120/s1, Figure S1: The expression of GLI2, PRR11, and SKA2 in H520 cells treated with GANT-61 or GLI1-siRNA was detected using Western blot; Figure S2: GANT-61 suppressed cellular proliferation and colony formation of LSCC cells; Figure S3: GANT-61 was more effective than GDC-0449 in reducing the survival of SK-MES-1 cells; Figure S4: The expression of NF-Y in H520 cells (A) and H226 cells (B) treated with GANT-61 was detected using Western blot; Table S1: Primers used for RT-PCR analysis; Table S2: two gene lists separately correlated with PRR11 expression and SKA2 expression in the LSCC cohort of TCGA; Table S3: GSEA reports of the PRR11-SKA2 correlated gene set in the LSCC cohort of TCGA; Table S4: The mRNA expression profiles of H226 cells treated with GANT-61 or DMSO detected by RNA sequencing; Table S5: The gene set which was downregulated in RNA sequencing and correlated with the PRR11-SKA2 gene pair.
