# Supplementary material for: HEDGEHOG/GLI Modulates the PRR11-SKA2 Bidirectional Transcription Unit in Lung Squamous Cell Carcinomas
Source: Genes (Basel). 2021 Jan 19;12(1):120. doi: 10.3390/genes12010120 (PMC7833434; doi:10.3390/genes12010120)
Supplement: Supplementary file 1 [file genes-12-00120-s001.zip › Table S1.docx]

**Table S1. Primers used for RT-PCR analysis**

| Gene name | Primer sequences |
| --- | --- |
| GAPDH | F807：5′-ACCTGACCTGCCGTCTAGAA-3′  R1053：5′-TCCACCACCCTGTTGCTGTA-3′ |
| GLI1 | F3313：5′-CCCAATCACAAGTCAGGTTCCT-3′  R3396：5′-CCTATGTGAAGCCCTATTTGCC-3′ |
| GLI2 | F1341：5′-AGATTCTGAGCCAGCAGAGG-3′  R1502：5′-TGGTGTCACTCAGACAGTTGC-3′ |
| PTCH1 | F2030：5′-GGCAGCGGTAGTAGTGGTGTTC-3′  R2220：5′-TGTAGCGGGTATTGTCGTGTGTG-3′ |
| E2F1 | F1056： 5′-CATCCCAGGAGGTCACTTCTG-3′  R1200：5′-GACAACAGCGGTTCTTGCTC-3′ |
| PRR11 | F566： 5′-GACTTCCAAAGCTGTGCTTCC-3′  R743： 5′-CTGCATGGGTCCATCCTTTTT-3′ |
| SKA2 | F198：5′-ACT TTGTATGCCCGCTTTAA-3′  R355：5′-GCCGCAGTTTTCTCTTCTTT-3′ |
| BIRC5 | F44：5′- AGGACCACCGCATCTCTACAT -3′  R161：5′- AGGACCACCGCATCTCTACAT-3′ |
| NCAPG | F114：5′-GAGGCTGCTGTCGATTAAGGA-3′  R228：5′-AACTGTCTTATCATCCATCGTGC-3′ |
| CCNB2 | F19：5′-CCGACGGTGTCCAGTGATTT-3′  R198：5′-TGTTGTTTTGGTGGGTTGAACT-3′ |
| BUB1 | F776：5′-ACAATCAACGGAGAAAGCATGA-3′  R915：5′-CTCCACCACCTGATGCAACT-3′ |
